# Persistence of Intracellular Bacterial Pathogens—With a Focus on the Metabolic Perspective

**DOI:** 10.3389/fcimb.2020.615450

**Published:** 2021-01-14

**Authors:** Wolfgang Eisenreich, Thomas Rudel, Jürgen Heesemann, Werner Goebel

**Affiliations:** ^1^ Department of Chemistry, Chair of Biochemistry, Technische Universität München, Garching, Germany; ^2^ Chair of Microbiology, Biocenter, University of Würzburg, Würzburg, Germany; ^3^ Max von Pettenkofer-Institute, Ludwig Maximilian University of Munich, München, Germany

**Keywords:** persistence, mechanisms of persister formation, intracellular bacterial pathogens, stress conditions, ATP-DnaA complex, DNA replication initiation

## Abstract

Persistence has evolved as a potent survival strategy to overcome adverse environmental conditions. This capability is common to almost all bacteria, including all human bacterial pathogens and likely connected to chronic infections caused by some of these pathogens. Although the majority of a bacterial cell population will be killed by the particular stressors, like antibiotics, oxygen and nitrogen radicals, nutrient starvation and others, a varying subpopulation (termed persisters) will withstand the stress situation and will be able to revive once the stress is removed. Several factors and pathways have been identified in the past that apparently favor the formation of persistence, such as various toxin/antitoxin modules or stringent response together with the alarmone (p)ppGpp. However, persistence can occur stochastically in few cells even of stress-free bacterial populations. Growth of these cells could then be induced by the stress conditions. In this review, we focus on the persister formation of human intracellular bacterial pathogens, some of which belong to the most successful persister producers but lack some or even all of the assumed persistence-triggering factors and pathways. We propose a mechanism for the persister formation of these bacterial pathogens which is based on their specific intracellular bipartite metabolism. We postulate that this mode of metabolism ultimately leads, under certain starvation conditions, to the stalling of DNA replication initiation which may be causative for the persister state.

## Introduction

### Bacterial Survival Strategies

Bacteria have evolved several strategies by which subpopulations are able to survive life-threatening conditions that are lethal for most members of bacterial populations. The best characterized strategies are: (a) formation of endospores (Higgins and Dworkin, 2012; Hutchison et al., 2014; Moir and Cooper, 2015), observed among Gram-positive bacteria, especially in the Bacillales and Clostridiales orders (all belonging to the phylum Firmicutes), (b) formation of exospores (Ohnishi et al., 2002; Sigle et al., 2015; Jones and Elliot, 2018) mainly found among members of the Actinomycetales, (c) formation of persister cells (Lewis, 2007; Balaban et al., 2013; Harms et al., 2016; Fisher et al., 2017; Radzikowski et al., 2017; Van Den Bergh et al., 2017; Kim J. S. et al., 2018; Wilmaerts et al., 2019) occurring in most bacteria, and (d) formation of the (apparently) related viable but not-culturable cells (VBNCs) (Oliver, 2010; Li et al., 2014; Ramamurthy et al., 2014). In all four survival states, metabolism and cell division either stop completely (as in case of endospores) or are at least highly reduced (as in case of persistent bacterial cells and VBNC). Common to all of these survival states is the ability of the “dormant” cells to rewake under favorable conditions and the unaltered genotype compared to the original cell population. It has been argued that the entry of a subset of cells into these survival states and their subsequent resuscitation might be a “bet-hedging” strategy allowing bacterial populations in general to withstand fluctuating environments (Grimbergen et al., 2015).

While the molecular mechanisms leading to spore formation are rather well characterized (Higgins and Dworkin, 2012; Paredes-Sabja et al., 2014; Setlow, 2014; Tan and Ramamurthi, 2014; Moir and Cooper, 2015), the underlying mechanisms causing bacterial persistence and the VBNC state are far from being fully understood (see below). The persistence of bacteria was first described in 1944 (Bigger, 1944) as a penicillin-insensitive, not inheritable state of a small subpopulation observed in an isogenic staphylococcal population. After antibiotic removal, the surviving bacterial cells started dividing again at normal growth rates generating again a similar fraction of persister cells upon renewed antibiotic treatment.

For bacterial pathogens, the persister state is most frequently observed after treatment with bactericidal antibiotics (Gollan et al., 2019; Bakkeren et al., 2020). It is of particular medical importance, apparently linked to persistent and chronic infections (Fauvart et al., 2011; Conlon, 2014; Kester and Fortune, 2014; Paredes-Sabja et al., 2014; Van Den Bergh et al., 2017; Jung et al., 2019). Recently, operational definitions and guidelines for *in vitro* studies of bacterial persistence have been reported (Balaban et al., 2019). According to these operational definitions, antibiotic tolerant bacteria are characterized by an exponentially slower killing rate in contrast to persisters which show a typical biphasic killing rate in comparison to that of antibiotic treated susceptible bacteria.

### Generation of Bacterial Persister and VBNC Cells

Antibiotic-persistent subpopulations can be detected in bacterial populations even when growing under favorable culture conditions. Upon treatment with high doses of (especially bactericidal) antibiotics, the large majority of the population is killed and a small persister subpopulation already preformed stochastically is then selected by the antibiotic treatment. However, there is evidence that antibiotic stress as well as other stress conditions (discussed in more detail below) enhance persister formation (Ayrapetyan et al., 2015; Nierman et al., 2015; Harms et al., 2016; Mok and Brynildsen, 2018). Regardless of the persistence-triggering condition, in all cases the formed persisters can be resuscitated when the stressors are removed and normal growth conditions are restored.

The VBNC state, first reported by Xu and collegues (Xu et al., 1982), is also observed as stress-surviving bacterial subpopulations and seems to be closely related to the persister state. VBNC cells and persister cells share many features and appear to co‐exist in the same bacterial population (Orman and Brynildsen, 2013; Ayrapetyan et al., 2015; Goncalves and De Carvalho, 2016; Zhao et al., 2017). Indeed, both persister and VBNC cells occur together in biofilms associated with infections (Spoering and Lewis, 2001; Li et al., 2014; Conlon et al., 2015). But unlike persister cells, VBNC cells have transiently lost the ability to grow in standard culture media. They can, however regain culturability in special complex media (Oliver, 2005). Some authors claim that VBNC cells may not represent a separate cell phenotype (Kim J. S. et al., 2018) and postulate that the term VBNC should be replaced with persister cells.

### Persister Formation of Intracellular Bacterial Pathogens

Formation of persister cells has been observed for all bacterial pathogens, including IBPs (Mulcahy et al., 2010; Li et al., 2014). Persister formation appears to be largely responsible for the recalcitrant chronic infections caused by *Mycobacterium tuberculosis* and *Chlamydia* species (Fauvart et al., 2011; Cohen et al., 2013; Srinivas et al., 2020). However, the mechanism(s) causing persister formation of IBPs when replicating in mammalian host cells is poorly understood.

After internalization by mammalian phagocytic or non-phagocytic host cells, most IBPs normally replicate in specifically modified endo(phago)somal compartments, called pathogen-containing vacuoles (PCV) or inclusions (in case of *Chlamydia*). These IBPs are termed “vacuolar IBPs”. Some IBPs termed “cytosolic IBPs” escape into the host cells’ cytosol and proliferate there (Ray et al., 2009; Pizarro-Cerda et al., 2016; Martinez et al., 2018; Omotade and Roy, 2019).

The replication rate of “cytosolic IBPs” is normally considerable higher than that of the “vacuolar IBPs”, presumably due to the better supply with essential nutrients in the cytosol. On the other hand, the vacuolar compartments may provide better protection against host innate immune attacks (Weiss and Schaible, 2015; Kim J. K. et al., 2018). In the context of persistence, it is interesting to note that persistent IBPs have been found exclusively in vacuolar compartments (see below), suggesting that these intracellular niches offer a better environment to reach the persistence state.

However, in both host cell compartments IBPs replicate in metabolically better defined environment than extracellular bacterial pathogens. The substantial progress over the last years in understanding the intracellular metabolism of IBPs and their adaptation to the metabolism of the host cells (Grubmüller et al., 2014; Eisenreich et al., 2017; Thompson et al., 2018; Best and Abu Kwaik, 2019; Eisenreich et al., 2019) may help to consider persistence of these bacterial pathogens from a metabolic point of view.

The focus of this review will be on persister formation of IBPs. First, we summarize the known bacterial factors and stress responses that have been associated with persister formation mainly in model bacteria (especially *Escherichia coli)*. The possible impact of this knowledge on persistence of IBPs will be considered. Then, we will discuss the specific metabolic states of IBPs when growing within host cells which may favor persister formation. This discussion is based on the concept of bipartite metabolism which appears to be a rather common strategy for IBPs (Grubmüller et al., 2014; Eisenreich and Heuner, 2016; Häuslein et al., 2016; Häuslein et al., 2017a; Mehlitz et al., 2017; Best and Abu Kwaik, 2019). Indeed, this metabolic approach along with its impact on DNA replication initiation and cell division may explain the long-term survival states (persistence, VBNC) observed in subpopulations of IBPs mainly as a continuum of intracellular metabolic alterations. We are aware that this approach neglects possible immunological aspects, e.g., the ability of persistent bacterial pathogens to avoid their elimination by innate and/or adaptive immune responses (Ulrichs and Kaufmann, 2002; Sabbagh et al., 2018; Gupta et al., 2019).

## Identified Bacterial Cellular Factors and Pathways Enhancing Persister Formation

### General Aspects

As mentioned above, bacterial persistence has been discovered 75 years ago, but the molecular mechanism(s) of persister formation is (are) still poorly understood (Kaldalu et al., 2016). Persisters can be apparently generated stochastically, probably due to the physiological heterogeneity of single cells in a bacterial population (Germain et al., 2015; Shan et al., 2017). The percentage of persisters in logarithmically growing cell cultures is small (<<1%), but significantly increased in the stationary-phase (Oliver, 2010). Persister formation is further triggered by various stress stimuli. A large number of molecular studies mainly performed with *E. coli* and a few other model bacteria, have identified different factors and stress response pathways that are apparently linked to persister formation. These include toxin-antitoxin (TA) systems (Maisonneuve et al., 2011; Balaban et al., 2013; Maisonneuve and Gerdes, 2014; Gerdes, 2016; Kedzierska and Hayes, 2016), oxidative stress response (Wu et al., 2012), RpoS-mediated general stress response (Mok et al., 2015; Liu et al., 2017; Mok and Brynildsen, 2018), stringent response together with the alarmone guanosine tetra-/pentaphosphate [(p)ppGpp] (Korch et al., 2003; Germain et al., 2015; Liu et al., 2017), DNA damage and SOS response (Dörr et al., 2009; Kreuzer, 2013; Völzing and Brynildsen, 2015), nutrient stress, and impaired energy production (Amato et al., 2013; Amato and Brynildsen, 2015; Shan et al., 2017; Mok and Brynildsen, 2018). All of these processes triggered by the corresponding stress stimuli lead to a significant increase of the persister fraction within bacterial populations.

It has been suggested that the toxin components of specific TA modules, more strongly expressed in some individual cells (possibly due to stochastic variation in ppGpp levels), are decisive factors for persister formation (Maisonneuve et al., 2013). However, strains with multiple deletions of genes encoding different TA modules (Maisonneuve et al., 2011) as well as ppGpp-negative strains (Maisonneuve et al., 2013) and *rpoS* deletion strains (Nguyen et al., 2011) still form persisters (though at reduced rates).

The discussed stress responses apparently involved in persister formation comprise complex regulatory networks that control the expression of multiple genes whose products are essential for coping with the stress conditions on the transcriptional, translational, and posttranslational level. In addition, many more genes (and in particular metabolic genes) than those directly involved in overcoming the stress situation are activated or repressed by the various stress regulators (Khil and Camerini-Otero, 2002; Jozefczuk et al., 2010; Yukihira et al., 2015; Christodoulou et al., 2018). Interestingly, transcriptome and metabolome studies indicate common metabolic pathways which are down- or up-regulated in a similar way by different stress conditions, such as temperature stress, oxidative stress, nutrient starvation, nutrient shifts or stationary phase (Jozefczuk et al., 2010). In addition, interactions and crosstalks exist between these regulons (Weber et al., 2005; Merrikh et al., 2009; Amato et al., 2013; Baharoglu et al., 2013; Leaden et al., 2018; Molina-Quiroz et al., 2018; Mitosch et al., 2019).

None of the described factors and pathways associated with persister formation converts the entire bacterial population into the persister state, although all (or at least most) cells in the population are subject to the respective stress stimulus and the subsequent response. This indicates that none of the above described stress conditions alone is the final cause of persistence and rather suggests that persistence is caused by the physiological heterogeneity triggered in bacterial populations under these conditions (Dhar and Mckinney, 2007; Gefen and Balaban, 2009). It is therefore more likely that a specific, yet unknown physiological state is responsible for persistence formation. This state appears to be reached stochastically in a few cells even of growing and unstressed bacterial populations (Amato et al., 2013; Radzikowski et al., 2017). The described stress conditions then stabilize and enhance this physiological state.

### Specific Stress Conditions Enhancing Persister Formation in Model Bacteria and IBPs

#### Bacterial Toxin/Antitoxin Modules and Their Association With Persistence

Bacterial TA modules are composed of a toxin and an antitoxin component that neutralizes the toxin. At least four different types of TA modules have been identified based on the function of the antitoxin (Yang and Walsh, 2017; Harms et al., 2018). Type I and especially type II TA modules, most widely distributed among prokaryotes (Gerdes et al., 2005; Fozo et al., 2008; Leplae et al., 2011), have been shown to be involved in inducing persistence (Vazquez-Laslop et al., 2006; Rotem et al., 2010; Maisonneuve et al., 2011; Maisonneuve and Gerdes, 2014; Verstraeten et al., 2015; Gerdes, 2016; Page and Peti, 2016). In type I TA modules, the antitoxin is an antisense RNA which binds to the toxin-coding mRNA and blocks its translation (Fozo et al., 2008), whereas type II TA modules consist of toxin and antitoxin polypeptides that form an inactive complex (Gerdes et al., 2005; Gerdes, 2016). In type II TA modules, proteolytic degradation (usually by Lon or Clp proteases) of the antitoxin frees the active toxin. Degradation or depletion of the antitoxin may occur stochastically or in response to stresses and the unleashed toxin protein affects central cellular processes, including translation, DNA replication, cell division, and metabolism (Gerdes, 2016; Kedzierska and Hayes, 2016; Harms et al., 2018; Wilmaerts et al., 2019).

Notably, the contribution of type II TA modules to persister formation has been recently challenged (Harms et al., 2017a; Goormaghtigh et al., 2018). Goormaghtigh and colleagues (Goormaghtigh et al., 2018) provided evidence that an *E. coli* K-12 mutant strain lacking the 10 type II TA modules previously postulated to participate in persister formation produced similar levels of persisters as the wild-type bacteria in unstressed cultures and after exposure to antibiotics [but see also (Holden and Errington, 2018; Ronneau and Helaine, 2019)].

##### TA Modules in IBPs

Especially, type II TA modules have been found in most human bacterial pathogens including several vacuolar and cytosolic IBPs. An unusually large number of TA modules has been identified in *Salmonella enterica* serovars (24 TA modules including 4 type I and 19 type II) (Mcclelland et al., 2001; Di Cesare et al., 2016) and in *M. tuberculosis* (79 TA modules) (Slayden et al., 2018; Thakur et al., 2018). Type II TA modules are also present in *Bartonella* (Harms et al., 2017b), *Listeria monocytogenes* (Curtis et al., 2017; Kalani et al., 2018), *Shigella* serovars (Mcvicker and Tang, 2016), *Rickettsia* spp. (Socolovschi et al., 2013), and *Brucella* spp. (Heaton et al., 2012). Neither type I nor type II TA modules have been found in *Chlamydia* spp. and there is also no convincing evidence for the presence of such TA modules in *Coxiella*, *Francisella* and *Legionella* (Pandey and Gerdes, 2005; Leplae et al., 2011; Yamaguchi et al., 2011).

The contribution of TA modules to persistence of IBPs has been extensively studied in *S.* Typhimurium (Helaine et al., 2014; Di Cesare et al., 2016; Vandrisse et al., 2017; Rycroft et al., 2018) and *M. tuberculosis* (Korch et al., 2009; Albrethsen et al., 2013; Schuessler et al., 2013; Korch et al., 2015; Winther et al., 2016; Slayden et al., 2018). In *L. monocytogenes*, MazEF, a type II TA module which contributes to persister formation in many bacteria (Gerdes et al., 2005), obviously does not affect the persister formation upon treatment with antibiotics in lethal doses (Curtis et al., 2017).

#### The Stringent Response, the Alarmone (p)ppGpp, and the Association With Persister Formation

Next to TA modules, (p)ppGpp appears to play a major role in persister formation (Korch et al., 2003; Kim H. Y. et al., 2018). This alarmone is the molecular effector of the bacterial stringent response which leads to an extensive transcriptional reprogramming and to metabolic changes in response to nutrient deprivation (Potrykus and Cashel, 2008). In *E. coli* (and all members of the gamma-proteobacteria), RelA and SpoT synthesize (p)ppGpp (in the following termed only ppGpp) from GTP and GDP, whereas in *Bacillus subtilis* and many other bacteria, a single enzyme (Rel or Rsh) is responsible for this activity (Mittenhuber, 2001). Upon amino acid starvation, the uncharged tRNAs activate the ribosome-associated RelA to synthesize ppGpp, whereas carbohydrates and fatty acid starvation stimulate ppGpp synthesis by the cytoplasmic SpoT (Xiao et al., 1991; Seyfzadeh et al., 1993). In a complex with DksA, ppGpp binds to RNA polymerase and inhibits transcription initiated from stable RNA (i.e., rRNA and tRNA) promoters and upregulates transcription of amino acid biosynthesis and stress response genes (Potrykus and Cashel, 2008; Dalebroux and Swanson, 2012; Hauryliuk et al., 2015).

As mentioned above, a link between ppGpp and persister formation was first shown by Korch et al. (2003) and confirmed by other studies (Amato et al., 2013; Bokinsky et al., 2013; Germain et al., 2013; Maisonneuve et al., 2013; Kim H. Y. et al., 2018) demonstrating that increased ppGpp levels result in growth arrest and increased persistence.

Interestingly, ppGpp is also a regulator especially for type II, but also for type I TA modules (Maisonneuve et al., 2013; Maisonneuve and Gerdes, 2014; Verstraeten et al., 2015; Tian et al., 2017). For type II TA modules, increased production of ppGpp activates the Lon protease-dependent antitoxin degradation and the released toxin component appears to increase the generation of persisters by blocking central cell processes (see above). This ppGpp-mediated activation of type II TA modules has become a widely accepted model for persister formation that has, however, also come under critical debate recently (Chowdhury et al., 2016a; Shan et al., 2017; Maisonneuve et al., 2018). Transcriptional control by ppGpp has also been shown for the type I TA module HokB/SokB (Verstraeten et al., 2015; Wilmaerts et al., 2018) as outlined below in more detail.

##### Association of Stringent Response and ppGpp With Persister Formation in IBPs

According to Kegg Data Base, all IBPs except the obligate intracellular pathogens *Chlamydia* and *Rickettsia* spp. produce RelA or a RelA/SpoT-like enzyme (Rsh), and are able to synthesize ppGpp. SpoT is present as separate enzyme in IBPs belonging to the gamma-proteobacteria, i.e., *Shigella*, *Salmonella*, *Francisella*, *Legionella*, and *Coxiella*, but is absent in *Chlamydia* spp. and *Rickettsia* spp. (Mittenhuber, 2001; Clark et al., 2011). In *L. monocytogenes*, three genes code for ppGpp synthetases: one bi-functional RSH enzyme and two small synthases (Natori et al., 2009). *M. tuberculosis* carries a gene encoding RelMtb, a bifunctional RelA/SpoT homolog that modulates synthesis and hydrolysis of ppGpp during the stringent response (Avarbock et al., 1999; Hogg et al., 2004). A single RelA/SpoT-like Rsh enzyme has also been identified in *Brucella* (Dozot et al., 2006).

The involvement of ppGpp in persister formation of IBPs has been suggested for *S. enterica* und *M. tuberculosis* (Helaine et al., 2014). Helaine and coworkers reported that *Salmonella* living within macrophage (MP) vacuoles are exposed to potentially stressful conditions that induce the expression of 14 type II TA modules in a ppGpp/Lon-dependent manner, and this event apparently plays an important role in the formation of persister cells. It has also been shown that, in *S.* Typhimurium, persistence triggered by ShpAB (also a type II TA module with Lon-dependence) also occurs in a *relA* mutant, i.e., in the absence of ppGpp synthesis (Slattery et al., 2013). One should keep in mind, however, that the involvement of ppGpp in persister formation is clearly not restricted to its role in the activation of TA modules (Liu et al., 2017) (see also below).

In *M. tuberculosis*, the single Rsh enzyme (RelMtb), responsible for ppGpp production, is required for the long term survival under *in vitro* starvation conditions (Primm et al., 2000). Dahl and colleagues (Dahl et al., 2003) reported that RelMtb is critical for the successful establishment of persistent infection in mice by altering the expression of antigenic and enzymatic factors that may contribute to successful latent infection. Stringent response (involving ppGpp) mediates persistence in *M. tuberculosis* (Chuang et al., 2015). *M. tuberculosis* strains expressing a mutant ppGpp synthetase (RelMtb) are unable to persist in mice, also demonstrating that the RelMtb activity is required for maintaining mycobacterial titers during chronic infection (Weiss and Stallings, 2013). A RelMtb mutant does not slow down replication during nutrient starvation and carries out a similar metabolism as the wild-type strain in nutrient-rich media (Dutta et al., 2019). Furthermore, it has been reported (Wayne and Lin, 1982; Dutta and Karakousis, 2014) that in *M. tuberculosis* cultures growing under optimal conditions, translationally dormant cells pre-exist as a small subpopulation and that part of these pre-existing persisters are RelMtb-overexpressing cells (Srinivas et al., 2020). Interestingly, the involvement of ppGpp in persister formation seems to be restricted to pathogenic mycobacteria (Bhaskar et al., 2018).

Concerning the involvement of ppGpp in *L. monocytogenes*, a study by Taylor and colleagues (Taylor et al., 2002) showed that a *relA* mutant, which was unable to accumulate ppGpp in response to amino acid starvation, was avirulent in a murine infection model (in contrast to the wild-type strain), indicating an essential role of stringent response for survival and growth of *L. monocytogenes* in this host. A link to persistence is not apparent in this study. In *Francisella*, the involvement of ppGpp in the regulatory network governing virulence gene expression has been established (Charity et al., 2009; Dean et al., 2009; Cuthbert et al., 2017), but again the possible role in persistence remains unclear.

The presence of persistent, clinically relevant *Legionella pneumophila* strains in different natural environments, often in close association with free-living amoebae and multispecies biofilms, is well documented (Berjeaud et al., 2016; Abu Khweek and Amer, 2018), but virtually nothing is known on the mechanisms causing this persistence. *L. pneumophila* requires synthesis of ppGpp in response to amino acid starvation to reach a state which allows the bacteria to escape from infected amoeba (Hammer and Swanson, 1999). During its intracellular life cycle in host MPs, *L. pneumophila* switches between a replicative and a transmissive state (Swanson and Fernandez-Moreira, 2002). In these host cells, ppGpp seems to be required for the transmission, since a *relA*/*spoT* mutant is killed during entry to and exit from MPs. Further work showed, however, that RelA (which senses amino acid starvation) is dispensable in MPs, whereas the hydrolase activity of SpoT (and hence hydolysis of ppGpp) is essential for the conversion of the bacteria from the transmissive to the replicative phase in MPs (Dalebroux et al., 2009). The authors conclude that the SpoT-mediated ppGpp degradation (monitoring fatty acid biosynthesis; see above) is necessary for this alternation in MPs. However, the question whether ppGpp also plays a role in persistence formation of *L. pneumophila*, remains unanswered (Abu Khweek and Amer, 2018).

In *Brucella* spp., stringent response is induced by nutrient stress *via* ppGpp that is synthesized by a single, bi-functional Rsh enzyme. *Rsh* deletion mutants of *Brucella suis* and *B. melitensis* show an altered morphology and a reduced survival rate under starvation conditions in cellular and murine infection models (Dozot et al., 2006). ppGpp-Dependent cross-talk between nutrient, oxidative, and low-oxygen stress responses was demonstrated suggesting an important role of ppGpp in the adaptation of *Brucella* to the host (Hanna et al., 2013) and possibly in persistence and chronic infections (Ficht, 2003).

*Coxiella* and *Bartonella* spp. possess ppGpp synthases, but nothing is known on the possible participation of ppGpp in persister formation of these IBPs. *Chlamydia* spp. and *Rickettsia* spp. lack ppGpp synthases and hence are unable to produce ppGpp (Mittenhuber, 2001).

#### General Stress Response and Its Link to Persistence

In addition to the ppGpp-dependent stringent response, the general stress response (GSR) appears to be also linked to persistence (Boaretti et al., 2003; Hong et al., 2012; Schellhorn, 2014; Tkachenko et al., 2014; Harms et al., 2016; Trastoy et al., 2018). In *E. coli* (and related bacteria), GSR depends on the sigma factor S (RpoS). RpoS governs the expression of many stationary-phase-inducible genes in *E. coli* (Hengge-Aronis, 1996; Battesti et al., 2011) and the entry into the stationary phase is known to lead to increased persister formation (Wood et al., 2013). A variety of environmental stress conditions can also induce GSR, including nutrient deprivation, variations of temperature, biofilm production, high pH, oxidative stress, and hyperosmolarity. GSR is also connected with ppGpp and TA modules: ppGpp stimulates the accumulation of RpoS (Brown et al., 2002; Hirsch and Elliott, 2002; Dalebroux et al., 2010). Antitoxins of certain TA modules repress the expression of RpoS. However, upon stress, the antitoxins are degraded and RpoS expression is induced (Wang et al., 2011; Wang and Wood, 2011; Hu et al., 2012).

##### GSR in IBPs and Its Link to Persistence

Among the IBPs, GSR is controlled by RpoS in the gamma-proteobacteria *Shigella*, *Salmonella*, *Legionella*, and *Coxiella*, and the involvement of RpoS in formation of persistence has been suggested for salmonellae and shigellae (Trastoy et al., 2018). In *L. monocytogens*, GSR is regulated by the alternative sigma factor SigB (Mittenhuber, 2002), similar as in *B. subtilis* and a small group of other Gram-positive bacteria (Hecker and Volker, 2001). SigB is involved in the survival of both saprophytic and host-associated stresses by *L. monocytogenes* (Dorey et al., 2019). A SigB-related factor is also present in *M. tuberculosis* (Mittenhuber, 2002), where it plays a major role in determining the level of tolerance to several drugs and the amount of persisters surviving drug treatment (Pisu et al., 2017).

GSR in alpha-proteobacteria, such as *Rickettsia*, *Bartonella* and *Brucella*, is controlled by a cascade including the alternative sigma factor αEcfG (also termed σE1 or RpoE1), the anti-sigma factor NepR, and the anti-anti-sigma factor PhyR (Fiebig et al., 2015; Francez-Charlot et al., 2015). In *Brucella*, these factors control transcription of approximately 100 genes involved in persistence in a BALB/c mouse chronic infection model (Kim et al., 2013; Willett et al., 2016). To our knowledge, there are no reports on a possible involvement of GSR-mediated regulation cascade in persister formation of *Bartonella* and *Rickettsia*. The obligate intracellular *Chlamydia* lacks all of these GSR-mediating sigma factors.

#### Oxidative Stress, Reactive Oxygen Species, Oxygen Stress Response, and the Links to Persistence

The connection between persister formation and oxidative stress, the subsequent increased reactive oxygen species (ROS) production, and the oxidative stress response (OSR) thereby induced, has been extensively described (Dörr et al., 2009; Möker et al., 2010; Vega et al., 2012; Wu et al., 2012; Wang et al., 2017). Augmented ROS production alters the membrane potential and causes damage of proteins, lipids, and nucleic acids (in particular DNA) with a strong impact on persister formation (Wang et al., 2017).

In *E. coli*, an increased ROS level induces the transcription factors SoxRS and OxyR that are primarily involved in the expression of antioxidant activities. But SoxRS can also induce the expression of the AcrAB-TolC multidrug-resistant pump causing extrusion of antibiotics. As consequence, a larger fraction of cells become persisters in the presence of antibiotics (Wu et al., 2012).

Increased persister subpopulations are also observed upon treatment of bacterial populations with bactericidal antibiotics (Kohanski et al., 2007; Kreuzer, 2013; Belenky et al., 2015; Kawai et al., 2015). Bactericidal antibiotics—besides blocking their primary targets—lead to downstream effects including metabolic changes accompanied with increased production of ROS (especially hydroxyl radicals) which, as already mentioned above, damage essential cellular components, ultimately causing cell death. In line with this assumption is the finding that bacteriostatic antibiotics, which recognize the same primary targets as the bactericidal antibiotics, do not trigger ROS production (Kohanski et al., 2007).

The enhanced persister formation arising upon treatment of a bacterial population with bactericidal antibiotics is apparently connected with this oxidative stress and the subsequent OSR leading to several response reactions that favor persistence (Walawalkar et al., 2016; Tosic-Pajic et al., 2017).

IBPs frequently encounter oxidative stress during infection. MPs—host cells for most IBPs—generate ROS and reactive nitrogen species (RNS) upon activation. Thus, ROS and subsequent OSR could also contribute to persistence (and possibly) chronic infections of IBPs. However, convincing experimental data are missing to support this assumption.

#### DNA Damage-Induced SOS Response and Its Link to Persistence

The association of persister formation to SOS response induced by DNA damage has been primarily studied in *E. coli*. The SOS pathway is crucially involved in the repair of DNA damage in bacteria (Kreuzer, 2013). The key regulators controlling the SOS network are LexA and RecA. Mutants lacking the *lexA* or the *recA* gene are more susceptible to quinolones and exhibit significantly reduced persistence in presence of these antibiotics (Dörr et al., 2009; Fung et al., 2010; Wu et al., 2012), while the constitutive expression of these genes strongly enhances persistence under these conditions (Dörr et al., 2009). These results suggest that persistence triggered by quinolones is influenced by the ability of the bacterial cell to repair DNA damage.

A link between SOS response and specific TA modules has also been demonstrated. Deletion of the SOS-inducible TisAB pair causes high reduction of persister cells; however, deletion of other LexA-box-containing TA pairs has no effect on persister formation (Dörr et al., 2010; Lewis, 2010). TisB is a membrane peptide that causes a decrease of the proton motive force and ATP levels. The resulting ATP depletion could therefore be also responsible for the SOS-induced persister formation by the TisAB TA module (Unoson and Wagner, 2008; Lewis, 2010; Shan et al., 2017). These examples show the complex interactions of the various stress conditions and the resulting cellular responses ultimately causing persister formation.

#### Involvement of the GTPase Obg in Persistence

Obg (also known as ObgE and CgtA) is a highly conserved GTPase present in all bacteria. It appears to function as a regulator for fundamental cellular processes such as ribosome maturation, DNA replication and chromosome segregation (Sikora-Borgula et al., 2002; Sikora et al., 2006; Persky et al., 2009; Kint et al., 2014). Obg has also been found to be central in controlling persistence in *E. coli* and *Pseudomonas aeruginosa* (Verstraeten et al., 2015). In *E. coli*, Obg-mediated persistence depends on ppGpp and the type I toxin HokB. An elevated ppGpp level induced by Obg leads to enhanced expression of the type I TA module HokB/SokB. The increased expression of the pore-forming HokB toxin results in a collapse of the membrane potential causing ATP leakage associated with persistence (Wilmaerts et al., 2018). All IBPs possess Obg-like proteins (**Table 1**), but there are no reports showing their involvement in persistence of IBPs.

**Table 1:** Presence and absence of factors in intracellular bacterial pathogens (IBPs) that were previously found to be associated with persistence.

IBP	TA	Stringent response			General stress response		Oxidative response		SOS response		Obg/CgtA
		RelA	SpoT	Rel (Rsh)	RpoS	SigB	OxyR	SoxR/SoxS	LexA	RecA	
**Vacuolar**											
*Salmonella* Typhimurium	**++**	**+**	**+**		**+**		**+**	**+/+**	**+**	**+**	**+**
*Mycobacterium tuberculosis*	**+++**			**+**		**+**	**+**	WhiB3	**+**	**+**	**+**
*Legionella pneumophila*	**(+)**	**+**	**+**		**+**		**+**	**−/−**		**+**	**+**
*Brucella mellitensis*	**+**			**+**	**(+)**		**+**	**−/−**	**+**	**+**	**+**
*Coxiella burnettii*	**(-)**	**+**	**+**		**+**		**+**	**−/−**	**-**	**+**	**+**
*Chlamydia trachomatis*	**-**	**-**	**-**	**-**	**-**		**-**	**−/−**	**-**	**+**	**+**
**Cytosolic**											
*Listeria monocytogenes*	**+**	**+**	**-**			**+**	**(-)**	**−/−**	**+**	**+**	**+**
*Shigella flexneri*	**++**	**+**	**+**		**+**		**+**	**+/+**	**+**	**+**	**+**
*Francisella tularensis*	**-**	**+**	**+**		**-**	**-**	**+**	**−/−**	**+**	**+**	**+**
*Rickettsia prowazekii*	**+**	**-**	**-**	**-**	**-**	**-**	**-**	−**/**−	**-**	**+**	**+**

(+) or (-) indicate unclear findings, while more than one + indicates the presence of multiple TA modules in the respective IBP. For more details, see also text.

## Metabolism and Persistence

### Facts and Hypotheses

Undoubtedly, metabolism plays a central role in initiating, maintaining and ending the persister state (Amato et al., 2013; Orman and Brynildsen, 2013; Amato et al., 2014; Prax and Bertram, 2014; Hartman et al., 2017; Radzikowski et al., 2017; Cabral et al., 2018). It has been shown that antibiotics (and the other stressors mentioned above) cause specific changes of the bacterial metabolism which may favor persister formation (Yang et al., 2017; Zampieri et al., 2017). In order to survive antibiotic treatment (or any other of the above described stress conditions), the persister cells must (a) shut down or silence essential physiological cell functions which antibiotics or the other stressors would irreversibly damage, (b) maintain viability during stasis, and (c) resume growth once the stress is lifted.

However, it is difficult to determine the specific metabolism of persister cells mainly due to their abundance, their transient nature, and their similar morphology in comparison to normal cells (Orman et al., 2015; Rowe et al., 2016). Therefore, the knowledge on the metabolism of bacterial persister state(s) is still fragmentary and sometimes even contradictory [see, e.g., (Leszczynska et al., 2013; Orman and Brynildsen, 2013; Kim and Wood, 2017)], although it is of great importance since it may offer novel strategies for the elimination of persisters (Allison et al., 2011; Fauvart et al., 2011; Kim et al., 2011; Radzikowski et al., 2017).

We will now focus on the presently known facts concerning the metabolism of persister subpopulations. Most of these data have been obtained by studies with few prototrophic model bacteria (mainly *E. coli*) which may limit their general validity. The major conclusions are: (a) Persistence often described as a “dormant state” represents a specific metabolically active state (Spoering et al., 2006; Prax and Bertram, 2014; Radzikowski et al., 2016). Despite the large decline in metabolic activities, the persister cells continue to produce energy, energize their membranes and produce a special set of proteins (Babin et al., 2017; Ayrapetyan et al., 2018). (b) The persistence-promoting metabolic processes occur stochastically in a small fraction even of actively growing and unstressed bacterial populations and are significantly enhanced in the presence of antibiotics, by nutrient starvation, in the stationary growth phase, and under the above mentioned stress conditions (Dörr et al., 2010; Radzikowski et al., 2016; Gutierrez et al., 2017; Meylan et al., 2017; Yang et al., 2017). (c) The persister state requires a suitable carbon and energy source (other than glucose) which allows a low flux through specific core metabolic pathways (Spoering et al., 2006; Amato et al., 2014; Cabral et al., 2018). (d) The tricarboxylic acid (TCA) cycle activity has been shown to play an important role in aminoglycoside antibiotic (tobramycin) susceptibility and tolerance of *P. aeruginosa* (Meylan et al., 2017): stimulation of the TCA cycle by fumarate activates cellular respiration and proton motive force. It leads to tobramycin uptake, high susceptibility and cell death, whereas shunting of the TCA cycle by stimulation of the glyoxylate cycle enhances tolerance against this aminoglycoside. (e) The ATP level is an important factor in persisters formation (Conlon et al., 2016; Radzikowski et al., 2016; Shan et al., 2017; Cameron et al., 2018). It has been even concluded that “stochastic variation in ATP is the main mechanism of persister formation” and “the decrease in ATP provides a satisfactory explanation for the drug tolerance of persisters” (Shan et al., 2017); (f) Central cell processes, including transcription, translation, DNA replication and cell wall synthesis, the major targets for most antibiotics (and eventually other stressors), are greatly slowed down or even turned off (Lewis, 2007; Hurdle et al., 2011; Kwan et al., 2013; Wood, 2017). (g) The metabolism of persisters may depend on the type of selection pressure, i.e., metabolism of persisters selected by ß-lactam antibiotics (cell wall inhibitors) is different from that of persisters selected by ciprofloxacin or nalidixic acid (both are gyrase inhibitors) (Cabral et al., 2018; Barrett et al., 2019). (h) An interesting, metabolism-based model has been proposed in which a specific low metabolic flux is the basis for establishing persistence, while other factors (e.g., various TA modules, ppGpp, RpoS) have a modulating and/or stabilizing function (Radzikowski et al., 2016).

### Metabolism of IBPs Within Host Cells and Possible Links to Persistence of IBPs

Regarding human IBPs, most work concerning the metabolism of persistent populations has been carried out with *M. tuberculosis*, *Chlamydia* spp. and *Salmonella* serovars where persistence appears to be a key factor for the often long lasting chronic infections. *M. tuberculosis* is a metabolically highly flexible pathogen able to adapt to the changing environments which this pathogen encounters during infection. Most studies dealing with metabolic aspects of persistent mycobacteria have been performed with various *in vitro* culture models and the murine *in vivo* model (Wayne and Lin, 1982; Dutta and Karakousis, 2014; Sohaskey and Voskuil, 2015). Persistence of *M. tuberculosis* in mice is promoted by the glyoxylate bypass (including isocitrate lyase, ICL) and deletion of the *icl* gene leads to reduced persistence. The *icl* mutant also shows decreased survival in activated but not in resting MPs (Mckinney et al., 2000). ICL, an enzyme involved in the mycobacterial glyoxylate and methylisocitrate cycle (Gould et al., 2006), is essential for the catabolism of lipids/fatty acids/cholesterol. Catabolism of host derived lipids including cholesterol is a major factor for the persistent state of *M. tuberculosis* (Bhusal et al., 2017; Bonds and Sampson, 2018).

The proper homeostasis of the oxido-reductive systems is likewise important for persistence and reactivation of *M. tuberculosis* (Kumar et al., 2011). *M. tuberculosis* possesses two succinate dehydrogenases (Sdh1 and Sdh2). Sdh1 but not Sdh2 is necessary for respiration through the electron transport chain under normoxic conditions. Sdh1 or Sdh2 appears to be essential for the respiratory adaptation to hypoxic environments leading to persistence (Hartman et al., 2014). Nitrate reduction also seems to serve a respiratory function upon a sudden shift of *M. tuberculosis* to hypoxia and the mycobacterial nitrate reductase activity is highly induced in the hypoxic state (Wayne and Hayes, 1998; Sohaskey, 2008). The genes for most of the functions important for the adaptation to hypoxic stress (and for persistence), including metabolic genes, such as those for nitrate reductase and enzymes involved in energy acquisition by alternative carbon substrates (e.g., fatty acids) and for synthesis of triacylglycerols, are under the control of the two-component system DosR-DosS (Park et al., 2003; Voskuil et al., 2003; Galagan et al., 2013). These and other studies, using *in vitro* and *in vivo* models of *M. tuberculosis* infection, show that the DosR regulon is an important control factor of persistence of *M. tuberculosis* under hypoxic conditions (Converse et al., 2009; Rustad et al., 2009; Leistikow et al., 2010; Liu et al., 2016).

Human-pathogenic *Chlamydia* species are obligate IBPs that cause a wide range of acute and chronic diseases. After having entered host cells (mainly non-phagocytic mucosal cells), these IBPs live in a vacuolar compartment, the “inclusion”. During the intracellular infection cycle, *Chlamydia* exist in two different forms. The reticulate body (RB) is the intracellular non-infectious, but proliferating form, which converts into the the elementary body (EB), the non-replicative, but infectious form. Under special conditions, such as treatment with interferon-gamma (IFN-γ) or penicillin, nutrient deprivation, or co-infection with *Herpes* viruses (Deka et al., 2006), RBs convert into persistent, nonreplicative particles, termed aberrant reticulate bodies (ARBs), which may re-convert into RBs and infectious EBs when the unfavorable conditions subside (Elwell et al., 2016; Witkin et al., 2017; Xue et al., 2017; Panzetta et al., 2018). Both the *Chlamydia* cells and the host cells undergo massive metabolic changes during the different conversions (Käding et al., 2014; Shima et al., 2018; Yang et al., 2019). In the persistent ARB state, *Chlamydia trachomatis* ceases to produce its major structural and membrane components (Witkin et al., 2017), but the still ongoing basic metabolic reactions in the ARBs remain largely unknown. In the infected host cells, IFN-γ activates the expression of indoleamine-2,3-dioxygenase 1 (IDO1), an enzyme that degrades tryptophan to kynurenine suggesting that the depletion of tryptophan blocks the normal chlamydial developmental cycle. Human-pathogenic *Chlamydia* species are unable to synthesize tryptophan. Restoration of tryptophan supply reverts the ARBs to infectious EBs (Beatty et al., 1993; Beatty et al., 1995), suggesting that biosynthesis of proteins containing multiple tryptophan residues may be blocked in ARBs (see below).

The metabolic requirement of persistent *Salmonella* has been mainly determined in an *in vivo* model of persistence whereby BALB/c mice were infected intravenously with a *S.* Typhimurium derivative that survived but hardly proliferated in the systemically infected mice. Persistent subpopulations could be isolated from liver and spleen which survived treatment with enrofloxacin, a fluoroquinolone highly efficient against this strain *in vitro* (Barat et al., 2012). Almost all tested *Salmonella* metabolic activities, including ATP synthesis driven by aerobic respiration, were dispensable in this persistence model demonstrating extensive resilience of persistent *Salmonella* against metabolic perturbations. However, inactivation of the biosynthesis of unsaturated fatty acids and cyclopropane fatty acids resulted in clearance of persisters to non-detectable levels in these organs. These fatty acids probably modify the fluidity of the cell wall. Upregulation of *cfa* genes (encoding cyclopropane fatty acid synthesis) was also observed in surviving *S. enterica* of different serotypes (including Typhimurium and Enteritides) upon long-term inoculation of poultry feed (Andino et al., 2014). This finding also suggests that the continued synthesis of these fatty acids is essential for long-term survival of *Salmonella* under harsh conditions. Isocitrate lyase appears to be required for *S.* Typhimurium persistence during chronic infection in mice but not during the acute phase of salmonellosis (Fang et al., 2005). This indicates that the glyoxylate shunt may play a critical role in the ability of *Salmonella* to persist in mammalian hosts and suggests that persistent *Salmonella* may depend on the utilization of fatty acids similar to *M. tuberculosis* (see above) and other intracellular pathogens (Monack et al., 2004). This conclusion is in line with the observation that *Salmonella* fail to persist in Parδ null mice (Eisele et al., 2013). The peroxisome proliferator-activated receptor delta (PPARδ) is a eukaryotic transcription factor essential for sustaining fatty acid metabolism (Odegaard et al., 2008) which is upregulated in *Salmonella*-infected MPs (Eisele et al., 2013).

Persistent *L. monocytogenes* strains are a major problem in food-industry (Ferreira et al., 2014; Abee et al., 2016), since severe infections in humans can be caused by food-borne strains that resist food processing steps (Lianou and Sofos, 2007) and even treatment with antimicrobials (Fox et al., 2011). Transcriptome analysis of such persistent strains show—compared to non-persistent strains—enhanced expression of the *pdu*, *eut* and *cob*-*cbi* operons (encoding enzymes involved in propandiol utilization, ethanolamine utilization, and cobalamine synthesis, respectively) (Fox et al., 2011). Whether these metabolic capacities may also play a significant role in persistent human listeriosis (Kleemann et al., 2009) remains an open question (Garsin, 2010).

Interestingly, the normally cytosolically replicating *L. monocytogenes* persist after infection of immunodeficient (SCID) mice in large vacuoles of liver granuloma MPs (Birmingham et al., 2008). Prolonged infection of (non-phagocytic) human hepatocytes and trophoblast cells also leads to bacteria enclosed in vacuoles. The switch from active cyctosolic replication to the resting vacuolar phase is correlated with a decreased accumulation of ActA at the bacterial surface (Kortebi et al., 2017). The authors argue that the formation of these *Listeria*-containing vacuoles could potentially enable the persistence in epithelial tissues. Although none of these two reports address the metabolic conditions in these vacuolar compartments and the metabolic activities of the persistent vacuolar bacteria, it is reasonable to assume that the conditions within the vacuoles (especially the metabolic conditions) are better suited for a persistent state than those in the host cell cytosol.

*Francisella tularensis*, another cytosolic IBP, can also re-enter into membrane-surrounded vacuoles (FCVs) after its extensive replication in the cytosol (Checroun et al., 2006) possibly to survive the more stressful conditions in the cytosol. To the best of our knowledge, there are no reports on IBPs persisting in the cytosol of host cells.

### Occurrence and Absence of Factors and Pathways Associated With Persistence in IBPs

As described above, several factors and pathways are associated with bacterial persistence. This correlation is mainly based on the observation that their expression enhances significantly the formation of persister cells whereas their deletion reduces persister formation. Most of these studies were performed with *E. coli* (see above). However, in contrast to *E. coli* and many extracellular bacterial pathogens, several human IBPs lack one or more of these factors and pathways, and *Chlamydia* spp. which belong to the most successful persisting bacterial pathogens, even lack all of them (**Table 1**). This fact suggests that other cellular processes must be responsible for persister formation in these IBPs. Common to all IBPs so far analyzed, including *Chlamydia*, is the (highly flexible) metabolic strategy that IBPs follow after internalization by mammalian cells which we termed “bipartite metabolism” (Grubmüller et al., 2014; Eisenreich et al., 2015).

### The Essential Features of Bipartite Metabolism

“Bipartite metabolism” (BM) describes a mode of bacterial metabolism that is carried out by many (possibly all) IBPs replicating within mammalian cells (Grubmüller et al., 2014; Eisenreich and Heuner, 2016; Häuslein et al., 2016; Chen et al., 2017; Häuslein et al., 2017a; Mehlitz et al., 2017; Best and Abu Kwaik, 2019). BM uses as major energy source various host-derived energy-rich carbon compounds that are less essential for the host cell than glucose. The withdrawal of these substrates from the host cell does not lead to the same negative consequences as withdrawal of glucose (e.g., autophagy and apoptosis). The suitable carbon compounds include mainly C_3_-metabolites such as pyruvate or glycerol, serine and cysteine that can be converted to pyruvate (Eylert et al., 2008; Alkhuder et al., 2009; Grubmüller et al., 2014; Puckett et al., 2014; Abu Kwaik and Bumann, 2015; Vanderven et al., 2015; Häuslein et al., 2016; Chen et al., 2017; Häuslein et al., 2017b; Mehlitz et al., 2017). Besides these C_3_-substrates, malate (used, e.g., by *Chlamydia*), fatty acids or cholesterol (used, e.g., by *M. tuberculosis*) are also suitable energy-rich components. These carbon sources are finally oxidized to acetyl-CoA, which enters the TCA cycle yielding important intermediates, as well as the electron carriers NADH and FADH_2_ which, as essential electron carriers, lead to ATP production by oxidative phosphorylation (OXPHOS). In the absence of a functional electron transfer chain (ETC), ATP can be also produced through substrate phosphorylation by conversion of acetyl-CoA *via* acetyl-phosphate to acetate. The latter ATP delivering step is present in all IBPs with the exception of *Chlamydia* spp. which can, however, import ATP from the host cell by ATP/ADP transporters (Tjaden et al., 1999).

*De novo* biosynthesis of IBP-specific cell components is normally restricted to those compounds that cannot be provided by the host cells. This concerns in particular cell wall components (e.g., meso-diaminopimelic acid, mDAP). For conducting these indispensable biosynthetic pathways, the IBPs import small amounts of host cell-derived glucose, glucose-6-phosphate or other carbohydrates that can be converted to glucose-6-phosphate with low energy consumption.

Thus, the concept of BM includes two distinct metabolic networks: a mainly catabolic part (P1) fed by the above mentioned C_3_ substrates which leads to the production of energy and some essential metabolites produced in the TCA cycle (e.g., oxaloacetate, α-ketoglutarate, and succinate) and another, more anabolic part (P2), fed mainly by intermediates of the upper part of the glycolytic pathway and the pentose phosphate pathway (PPP), which is essential for the indispensable anabolic components (mainly essential components for cell wall biosynthesis) that cannot be delivered by the host cell. The P1 and P2 networks interact depending on the physiological state of the host cell (Eisenreich et al., 2017; Eisenreich et al., 2019). For example, most of the energy-delivering carbon substrates mentioned above are glucogenic, i.e., in principle they are able to produce glucose by entering the gluconeogenic pathway. Indeed, most IBPs possess all genes required for the gluconeogenesis enzymes (exceptions are *Rickettsia* spp.). However, this mode of glucose production requires a disproportionally large amount of energy (e.g., glucose production from pyruvate needs six molecules of ATP) and gluconeogenesis is usually too expensive for carbohydrate production under intracellular conditions.

Under nutrient-rich conditions of the host cell, some IBPs, like *C. trachomatis*, *L. pneumophila* and *M. tuberculosis*, may convert suitable carbon substrates to storage products such as glycogen (Gehre et al., 2016), polyhydroxybutyrate (PHB) (Gillmaier et al., 2016; Häuslein et al., 2017b) or triacylglycerols (Maurya et al., 2018). These polymers can then be degraded to useful carbon/energy substrates under starvation conditions.

All studies carried out so far (Eylert et al., 2008; Grubmüller et al., 2014; Häuslein et al., 2016; Chen et al., 2017; Häuslein et al., 2017a; Mehlitz et al., 2017) indicate that, in IBPs, most low-molecular precursors for macromolecules, e.g., amino acids, nucleotides, fatty acids and vitamins, are imported from the host cells, which in turn obtain most of these nutrients from the intercellular space of host tissue. Major exceptions are, however, the three non-essential amino acids Ala, Asp, and Glu, which are efficiently synthesized *de novo* by all IBPs tested so far. Interestingly, these amino acids (in their D-forms) are either directly needed in considerable quantities for the synthesis of cell wall components (peptidoglycan and (lipo)teichoic acids) or act, like Asp, as a precursor of meso-diaminopimelate (mDAP), which also represents an important building block for peptidoglycan and is synthesized *de novo* by all IBPs except *Francisella*. The latter IBP probably uses Lys (which can again be obtained from the host cell) instead of mDAP for peptidoglycan synthesis. Thus, for carrying out intracellular replication, the IBPs import, in addition to the energy-delivering (e.g., glycerol) and anabolism-supporting (e.g., glucose-6P) carbon substrates, substantial amounts of low molecular metabolites from the host cell and restrict their anabolic activities to the biosynthesis of those components that cannot be provided by the host cells and, of course, to protein and nucleic acid biosynthesis.

Besides being an economic strategy for maintaining the metabolism of IBPs replicating in mammalian host cells, BM may also be advantageous for the expression of virulence factors that are essential for intracellular proliferation. Expression of these factors is often blocked (e.g by catabolite repression) when glucose is the major carbon source (Eisenreich et al., 2017). Furthermore, the metabolic flexibility provided by the BM strategy may lead to considerable metabolic heterogeneity in an IBP population during infection of mammalian host cells which may favor the formation of persister state(s). Indeed, metabolic heterogeneity in isogenic bacterial population has been extensively described (Ackermann, 2015; Sheik et al., 2016; Simsek and Kim, 2018) and its significance for persister formation discussed (Balaban et al., 2004; Jones and Lennon, 2010; Bald and Koul, 2013; Simsek and Kim, 2018).

### Metabolic States of the Host Cells and the Intracellular Bacteria That May Lead to Persister Formation

Ignoring immunological aspects, one can easily visualize two well-defined metabolic borderline states of the host cells that will either lead to proliferation or killing of the IBPs. In the first state, the host cells provide to the IBP a well-balanced supply of the above mentioned necessary nutrients. Under these conditions, all metabolites and sufficient energy will be available for the IBP to successfully carry out BM. This metabolic state will enable the IBP to build up its macromolecular structures and to perform efficient proliferation.

In the second state, the host cell can neither provide the main energy-delivering carbon source (in the following termed ECS) nor the carbon substrate supporting the indispensable anabolic processes (termed ACS) of the IBP. Under these conditions, the internalized IBP will come under nutritional stress and the resulting ATP shortage will ultimately lead to the stop of all macromolecular biosynthesis and the breakdown of the ATP-dependent DNA repair functions—and finally to the bacterial cell death.

Most differentiated cells, such as non-activated MP or epithelial cells have a reduced basic metabolism (resembling more the second metabolic host cell condition). This metabolic state provides probably not enough ECS and ACS to support efficient replication of internalized IBPs. However, most IBPs apparently express specific fitness factors that can activate the metabolism of the resting host cells converting them either into hospitable or sometimes also hostile states [recently reviewed by (Eisenreich et al., 2019)].

A typical example are the different metabolic activation events of resting MPs by Gram-negative bacterial pathogens: LPS of Gram-negative pathogens leads to classic M1 polarization of MPs which is characterized by a metabolic switch from the OXPHOS-driven metabolism (occurring at low level in resting MPs) to a Warburg-like metabolism with induced glucose uptake, aerobic glycolysis, lactate production, enhanced PPP and decreased TCA cycle activities (Krawczyk et al., 2010; O’neill, 2014; Kelly and O’neill, 2015). This metabolic state of the host cell could provide an appropriate nutrient supply to internalized IBPs and allow in principle bacterial replication. However, the production of oxygen and nitrogen radicals associated with the M1 state is deleterious for most IBPs (Eisele et al., 2013; Xavier et al., 2013a; Price and Vance, 2014). On the other hand, the alternative activation of MPs, induced by IL-4 which may be triggered in MPs by other bacterial factors leads to the anti-inflammatory M2 phenotype. The metabolism of M2 polarized MPs is characterized by enhanced fatty acid oxidation, OXPHOS, and thus increased intracellular levels of unconsumed glucose. This host cell metabolism appears to be favorable for IBP replication and has led to the assumption that M2 MPs could in general represent comfortable host cells for IBPs (Xavier et al., 2013b; Buchacher et al., 2015). However, the metabolic programs that can be induced in resting MPs (e.g., by IBPs) appear to be more diverse than just the M1 or M2 states (Murray and Wynn, 2011; Guilliams et al., 2014; Murray et al., 2014). There is experimental evidence that the metabolism of IBP-infected MPs clearly differs from that of M1 or M2 MPs (Götz et al., 2010; Gillmaier et al., 2012; Mehlitz et al., 2017). This metabolic variability of MPs may thus lead to a considerable metabolic heterogeneity in the MP population when exposed to IBPs. The obvious variability in bacterial numbers ranging from single to many bacteria per host cell observed when primary MPs are infected with IBPs (Sheppard et al., 2003; Thöne et al., 2007; Gillmaier et al., 2012) could be caused—at least in part—by the metabolic flexibility of these host cells.

**Figure 1:**
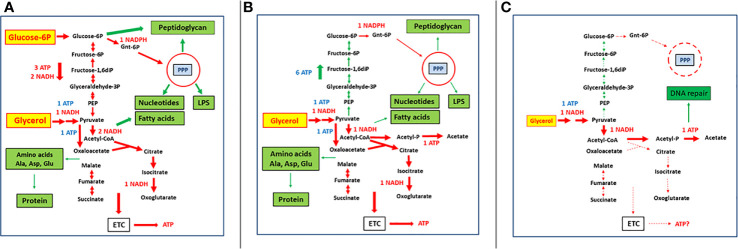
Bipartite metabolism of IBPs on the example of intracellular *Listeria monocytogenes* (Grubmüller et al., 2014; Eisenreich et al., 2017) showing various metabolic states and their possible significance for formation of persistence. **(A)** Host cell provides to intracellular *L. monocytogenes* a sufficient amount of glycerol as an energy source and a limited amount of glucose-6-phosphate used mainly for anabolic processes. **(B)** Host cell is unable to provide glucose-6-phosphate but can still provide a sufficient amount of glycerol which has now to be used by *L. monocytogenes* as energy source and for maintaining anabolic processes. **(C)** Host cell can only provide a limited amount of glycerol which is now mainly used by the intracellular bacteria as energy source to provide sufficient ATP for repair functions (mainly for DNA repair). Red arrows indicate mainly catabolic processes and green arrows anabolic processes. The thickness of the arrows indicates the strength of the processes under the given nutritional conditions. Dashed thin arrows indicate reactions that are no longer occurring or severely restricted under the given condition. Red-labelled ATP, NADH, and NADPH mean formation and blue ones consumption by the corresponding processes. See text, for further details.

The BM strategy of the IBPs likely contributes to metabolic heterogeneity of the intracellular bacteria. In the following, we consider several metabolic scenarios of the host cell which an incoming IBP may face and how the bacterial cell may adapt its own metabolism to the different metabolic states of the host cell—also with regard to possible persister formation:

Scenario 1: The host cell is able to provide sufficient amounts of the energy-delivering carbon substrate (ECS) and the anabolism-supporting carbon substrate (ACS) to the intracellular IBP (**Figure 1A**). Under these conditions, the IBP can replicate at optimal rates as long as the host cell tolerates the increasing number of IBPs. Then, the IBPs will be released from the host cells by different mechanisms or spread into neighboring host cells. It is unlikely that bacterial persister cells are formed under these conditions.

Scenario 2: The host cell can provide ACS, but not ECS. This is an unlikely scenario, since the catabolism of ACS by the host cell will always lead to ECS production. Besides, this scenario would resemble the growth conditions which the IBP face in a rich *in vitro* culture media where excess of ACS (especially glucose) blocks the synthesis of virulence factors that are necessary for the intracellular IBP life cycle.

Scenario 3: The IBP may receive from the host cell sufficient supply of ECS, but no ACS (**Figure 1B**). This situation can occur when the whole host cell population runs out of glucose. It may also happen stochastically when either a single host cell is depleted of ACS (e.g., glucose) or unable to take up ACS. If ECS is still available in sufficient quantity, the TCA cycle of the IBP can function and enough ATP is produced by oxidative and/or substrate phosphorylation to maintain at least all repair functions in the IBP. In case ECS is a glucogenic substrate (which is true for most C_3_-compounds serving as ECS), glucose (ACS substrate) may be produced by gluconeogenesis as long as there is enough ATP to keep this high energy-consuming pathway running. Under these conditions, the IBPs can still perform their own essential anabolic processes and can still multiply. Formation of persister cells is also less likely under these conditions.

Scenario 4: An additional shortage of ECS supply (this could again occur stochastically in single cells or in the entire host cell population) will lead to a continuous decrease of ATP production (**Figure 1C**). As a result, all high-energy-consuming anabolic processes, including protein, DNA, RNA and cell envelope synthesis, will gradually come to an halt. But even at low ECS concentration, low residual ATP synthesis is still possible by substrate phosphorylation through oxidation of the residual ECS to acetate. Indeed all IBPs (with the exception of *Chlamydia* spp.) have the capacity to convert ECS substrates *via* acetyl-CoA and acetyl-phosphate to acetate, thereby generating ATP. *Chlamydia*, missing this pathway, can however import ATP directly from the host cell (Schmitz-Esser et al., 2004). This residual ATP supply will still maintain necessary repair functions of macromolecules, especially of DNA (Dahan-Grobgeld et al., 1998), which is absolutely crucial for the viability of persister cells (Völzing and Brynildsen, 2015).

Under these ECS-limited conditions, various metabolic states can be anticipated which may lead to slow-growing and non-growing but surviving (persistent)? IBPs. Indeed, single cell analysis of IBP-infected host cells show a high heterogeneity of intracellular bacterial counts in *in vitro* and *in vivo* infection models, with most host cells containing few or often only one bacterial cell (Brown et al., 2006; Helaine et al., 2010; Watson and Holden, 2010; Gillmaier et al., 2012; Zuck et al., 2015; Dhar et al., 2016; Eisenreich et al., 2019).

## Does the Prevention of ATP-DNAA-DEPENDENT Initiation of DNA Replication Lead to Persister Formation?

### DnaA Is the Essential Initiator Protein for DNA Replication in Bacteria Including All IBPs

The above described metabolic scenarios 1–4 can occur in all IBPs with varying probability and scenario 4 could lead to persister formation even in those IBPs that lack most functions claimed to enhance bacterial persister formation (**Table 1**). This is the case in particular for human-pathogenic *Chlamydia* species that can nevertheless successfully form persistence.

A common cellular process to which all of the discussed persister-enhancing conditions appear to converge is the initiation step of DNA replication. This critical step in the bacterial cell cycle depends in bacteria, including all human IBPs, on the initiator protein DnaA, more precisely on ATP-DnaA, the initiation-active form (Sekimizu et al., 1988). For recent reviews, see (Katayama et al., 2017; Dewachter et al., 2018; Hansen and Atlung, 2018). Inhibition of ATP-DnaA formation results in a reversible stop of DNA replication initiation and cell division will subsequently stop. The resulting replication-terminated DNA is in a closed circular conformation which is less sensitive to damage (e.g., by oxidative stress induced by bactericidal antibiotics) than DNA with stalled elongation which always yields two open replication forks that are highly susceptible to damage (Hanna and Carl, 1975; Zyskind et al., 1977; Ikeda et al., 2012; Molina-Quiroz et al., 2018). In the following, the most important aspects of the ATP-DnaA dependent initiation step of DNA replication is shortly summarized. For further details on this complex field, see expert reviews (Leonard and Grimwade, 2010; Katayama et al., 2017; Hansen and Atlung, 2018; Leonard et al., 2019).

Initiation of DNA replication needs a critical amount of ATP-DnaA per oriC (the unique origin of chromosomal DNA) (Fuller et al., 1984; Morigen et al., 2003; Skarstad and Katayama, 2013; Hansen and Atlung, 2018). The initiation-active ATP-DnaA complex is inactivated shortly after initiation of DNA replication at oriC by hydrolysis of ATP to ADP resulting in an initiation-inactive ADP-DnaA complex (Keyamura and Katayama, 2011; Kasho and Katayama, 2013; Kasho et al., 2017). This process prevents that more than one round of DNA replication is initiated per cell cycle—at least at the low replication rates which is the case for IBPs growing in host cells. Re-initiation of DNA replication requires a precisely regulated amount of DnaA protein and sufficient ATP to form again an active ATP-DnaA complex at the newly formed oriC. The critical level of DnaA protein in the bacterial cell is essentially obtained by the rejuvenation of ADP-DnaA to DnaA and by *de novo* synthesis of DnaA (Castuma et al., 1993; Fujimitsu et al., 2009; Dewachter et al., 2018). Renewal of DnaA is achieved by several (seemingly bacteria-specific) mechanisms. For details, see recent reviews (Dewachter et al., 2018; Hansen and Atlung, 2018). The amount of *de novo* synthesized DnaA appears to be regulated on the transcriptional, translational and post-translational level. Transcription of the *dnaA* gene proceeds from two promoters, p1 and p2, which is auto-regulated, i.e., both promoters are inhibited by ATP-DnaA (Braun et al., 1985). Earlier studies claimed that high levels of the alarmone ppGpp also repress *dnaA* transcription, especially from the major promoter p2 (Chiaramello and Zyskind, 1990). More recent data show that ppGpp rather prevents replication initiation by blocking the introduction of initiation-promoting negative supercoils through inactivation of RNA polymerase by binding of ppGpp and thus preventing gene transcription and negative supercoiling of DNA (Kraemer et al., 2019). The *dnaA* transcript is rather unstable and has a low translation frequency (Bernstein et al., 2002) further limiting the synthesis of DnaA protein. Although DnaA is a rather stable protein, excess DnaA can be acetylated at a highly conserved lysine (K178) by the acetyltransferase YfiQ (and non-enzymatically by acetylphosphate) which prevents DnaA from binding to ATP and hence to oriC (Li et al., 2017). In *E. coli*, the level of DnaA acetylation correlates with the frequency of replication initiation and reaches a peak at the stationary phase, leading to inhibition of initiation. This regulatory step of DnaA is reversible and deacetylation is catalyzed by the deacetylase CobB (Zhang et al., 2016). In *Caulobacter crescentus*, it has been shown that carbon starvation and nutritional depletion which take place, e.g., in the stationary phase lead to Lon-dependent proteolysis of DnaA and decreased translation of the *dnaA* transcript, thus reducing the accumulation of DnaA protein below the critical level necessary for initiation of replication (Leslie et al., 2015). Athough ATP binds to DnaA with high affinity, a critical cellular ATP/ADP ratio is required to charge the *de novo* synthesized and rejuvenated DnaA protein to form initiation-active ATP-DnaA. Finally, the membrane fluidity and especially its content of acidic phospholipids also play an essential role in the formation of a functional initiation complex between oriC and ATP-DnaA together with some other proteins (Norris, 1990; Castuma et al., 1993; Saxena et al., 2013; Katayama et al., 2017).

**Figure 2:**
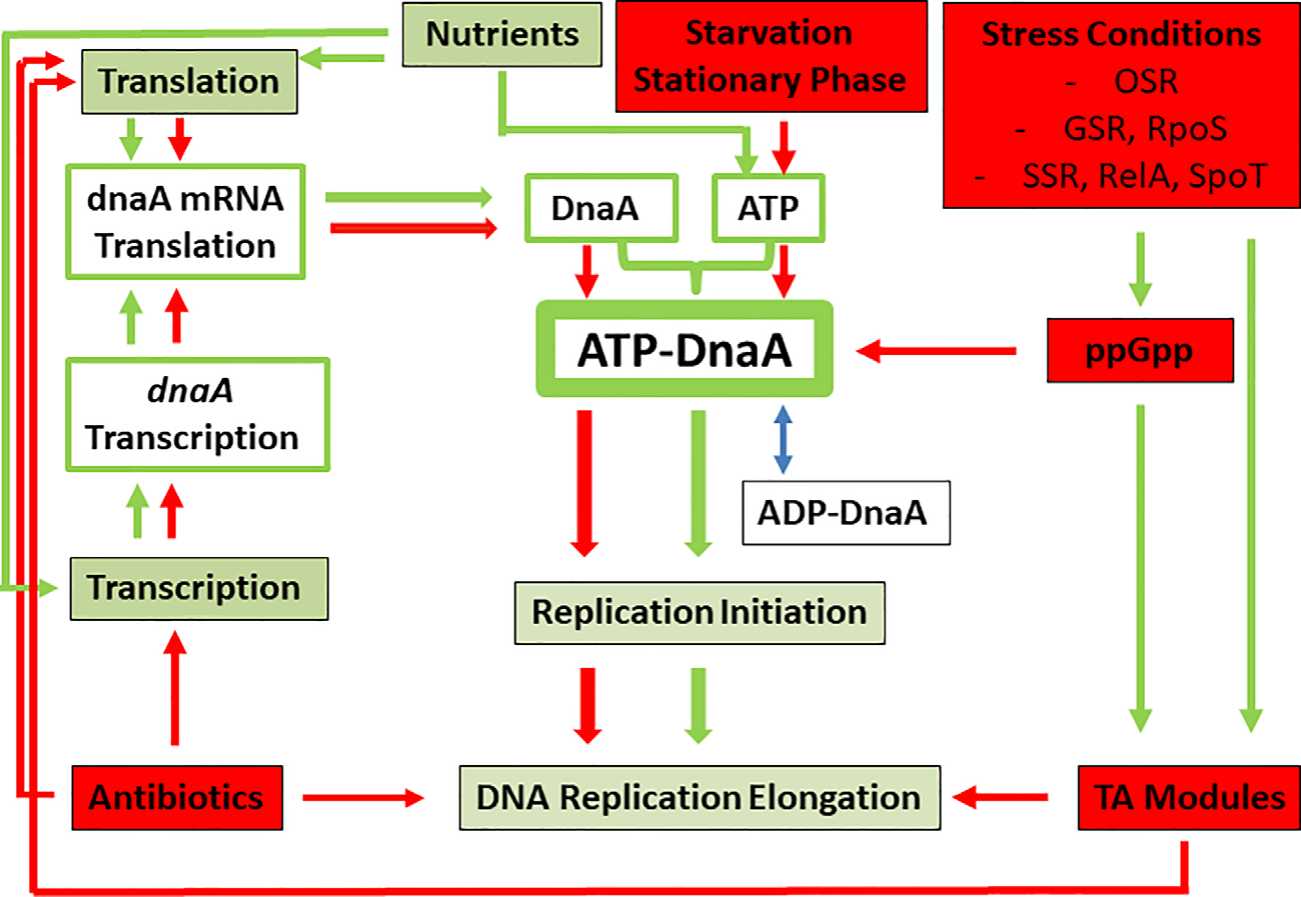
Model showing the major processes necessary for the generation of the DNA initiation-active ATP-DnaA complex (green boxes and green arrows) and the factors, conditions and pathways (red boxes and red arrows) leading to inhibition of ATP-DnaA complex formation. Note that the latter situations are also identical to those leading to persister formation. OSR, oxidative stress response; GSR, general stress response; SSR, stringent stress response, leading to the generation of ppGpp *via* RelA and SpoT or Rsh. See text, for further details.

The central components involved in initiation of DNA replication, i.e., DnaA, ATP and oriC, are common to bacteria (Wolanski et al., 2014). The processes listed below (*Stochastic Occurrence* to *ATP Limitation*) leading to reversible stop of DNA replication initiation (due to the failure to form an active ATP-DnaA/oriC complex) are similar to the above discussed cellular events that favor bacterial persistence. It is therefore intriguing to hypothesize that persistence is linked to or even caused by stalling of DNA replication initiation due to insufficient cellular concentration of DnaA and/or ATP, or the failure to form an active ATP-DnaA/oriC complex. In the following, we point out that conditions known to lead to persister formation also negatively affect the formation of the ATP-DnaA complex and hence prohibit the initiation of DNA replication (see also **Figure 2**).

#### Stochastic Occurrence

In normal (i.e., unsynchronized) logarithmically growing *E. coli* cultures, DNA replication is in all possible stages. Most cells are in an ongoing replication process and their DNA will contain two replication forks due to the bidirectioal chromosomal replication (we will call this population of actively DNA replicating cells: a-cells). In a few cells, the replication is terminated but not yet re-initiated (this cell population will be called t-cells). The ratio of a-cells/t-cells depends on the growth rate and the growth phase, i.e., it will be higher in nutrient-rich culture media than in nutrient-poor ones and lower in the stationary than in the logarithmic growth phase. We postulate that the persister subpopulation observed in growing, unsynchronized bacterial populations always originates from the t-cell subpopulation.

#### Presence of Antibiotics

DNA replication inhibitors, like the gyrase-inhibiting quinolone antibiotics (e.g., nalidixic acid or ciprofloxacin), will quickly stop replication of a-cells. The stalled replication forks of a-cells are highly sensitive to damage and if not rapidly stabilized, these damages will lead to cell death by different processes, including double-strand breaks (Kuzminov, 1995; Kohanski et al., 2007; Masai et al., 2010; Liu and Imlay, 2013). The t-cell population, on the other hand, has a more stable, in most cases covalently closed circular DNA conformation (Nordström and Dasgupta, 2001) that can re-initiate replication once the antibiotic stress is released (Dewar and Walter, 2017). Antibiotics acting as inhibitors of translation or transcription (such as aminoglycosides, chloramphenicol or rifampicin) do not directly interfere with the DNA replication machinery, i.e., they do not disrupt the elongation step. These antibiotics can inhibit, however, initiation of DNA replication by blocking *de novo* synthesis of DnaA (Lark, 1972; Skarstad and Katayama, 2013), which is (at least in part) required for re-initiation of DNA replication. Besides, the bactericidal representatives among this group of antibiotics strongly interfere with DNA replication by formation of ROS and other processes that are lethal for the a-cell but less for the t-cell population (Yamaki et al., 1986; Hiraga et al., 1990; Miller et al., 2004; Kohanski et al., 2007; Liu and Imlay, 2013). This means that these antibiotics, which, similar to the gyrase-inhibiting antibiotics, cause enhanced persister formation, also lead to an increased t-cell subpopulation by inhibiting initiation of DNA replication. On the other hand, antimicrobials causing cross-linking of DNA, e.g., mitomycin, which lead to death even of persister cells (Keren et al., 2012; Chowdhury et al., 2016b) will also be lethal to t-cells, since re-initiation of cross-linked DNA can no longer occur.

#### TA Modules

Although most toxin components of TA modules target translation, some of them, e.g., ParE, CcdB, FicT, interfere with DNA replication by inhibiting the gyrase activity (Harms et al., 2018) and hence may exert similar inhibitory effects on DNA replication as the above described quinolone antibiotics. However, the majority of the toxins target translation in different ways (Harms et al., 2018). Thus, similar to translation-inhibiting antibiotics, TA modules may also exert inhibitory effects on *de novo* synthesis of DnaA and, hence, on initiation of DNA replication.

#### Increased Production of ppGpp

Amino acid starvation and other stress conditions lead to enhanced synthesis of ppGpp which increases persister formation and is also accompanied by inhibition of replication initiation due to different mechanisms including inhibition of RNA polymerase by ppGpp (Zyskind and Smith, 1992; Schreiber et al., 1995; Kraemer et al., 2019).

#### ATP Limitation

The formation of initiation-active ATP-DnaA requires not only a critical amount of DnaA protein but also a critical level of cellular ATP (Dewachter et al., 2018). Besides, DNA damage suffered by the t-cell population (e.g., by ROS) has to be repaired before proper reinitiation can occur. This repair process requires ATP and in the absence of sufficient ATP even t-cells cannot be revived.

### Persistence Formation of IBPs and Its Possible Link to the DnaA-Dependent Initiation of DNA Replication

The formation of IBP persisters can also be explained by the above hypothesis. As outlined above, persister formation of all IBPs will be in general favored under intracellular (vacuolar) conditions which may reduce the metabolic activities and energy production of the IBPs (see **Figure 1C**) due to insufficient supply of essential nutrients from the host cell (Monack et al., 2004; Helaine et al., 2010; Helaine and Holden, 2013). It is likely that under these conditions the probability is increased that in some cells of an intracellular IBP population re-initiation of terminated DNA replication is prevented due to insufficient amount of ATP and/or DnaA protein, similar to cells in the stationary phase of *in vitro* growing bacterial cultures. We postulate that these IBP cells will become antibiotic-insensitive persisters.

Formation of persistence in IBPs on the basis of stalled replication initiation is outlined in the following more extensively on the example of *C. trachomatis*. This bacterium is an obligate IBP that lacks most factors and pathways that were previously associated with persister formation (**Table 1**). Yet, this pathogen is highly successful in persister formation.

As described above, *C. trachomatis* RBs enter the persister state when exposed to different conditions, such as treatment with IFN-γ or antibiotics including penicillin (Panzetta et al., 2018), azithromycin (Xue et al., 2017), and erythromycin (Clark et al., 1982), by amino acid starvation (Wyrick, 2010), or co-infection with *Herpes* viruses (Deka et al., 2006; Prusty et al., 2012). The persistent forms (ARBs) are viable, non-dividing and often enlarged cells that can revert to (RBs and) infectious EBs after removal of the persistence-inducing conditions (Panzetta et al., 2018).

ARBs generated in presence of IFN-γ carry out a generally reduced metabolism, slow down DNA replication, stop cell division, but continue to transcribe genes with different efficiencies (Ouellette et al., 2016), and show even enhanced synthesis of some proteins. The most striking ones among them are the chaperonins GroEL (Hsp60) and GroES (Witkin et al., 2017), and tryptophan synthase (TrpAB) (Belland et al., 2003). In the infected HeLa cells, IFN-γ reduces glycolysis, accompanied by the reduction of glucose transporter-1 (GLUT1) and of the hypoxia inducible factor-1a (HIF-1a) (Shima et al., 2018) leading to reduced ATP level in the host cells.

But in particular—as described above—IFN-γ induces indoleamine 2,3-dioxygenase (IDO). IDO strongly decreases the level of tryptophan in the host cell by degrading this amino acid to kynurenine thereby stopping the supply of tryptophan for intracellular *Chlamydia* which these bacteria are unable to synthesize. The ARBs formed under these conditions can readily be reactivated to normal infectious EBs when either IFN-γ is removed or tryptophan is added to the culture medium (Beatty et al., 1995; Muramatsu et al., 2016), suggesting that ARB formation is directly linked to the tryptophan deficiency.

Interestingly, proteins that continue to be synthesized or are even induced in the ARBs contain little or no tryptophan (e.g., GroES, GroEL, and TrpAB). Most other proteins contain on the average 1% tryptophan residues or more in their polypeptide chains (Ouellette et al., 2016) and, hence, their *de novo* synthesis will be reduced or even blocked. In this respect, the initiator protein DnaA is of particular interest, since it has to be (at least in part) *de novo* synthesized for initiating a new round of DNA replication. DnaA1 contains four Trp residues, three of which are within the first 50 amino acids of the 456 amino acid containing protein. It is therefore possible that—due to the IFN-γ induced tryptophan deficiency—*de novo* synthesis of DnaA may gradually cease, although the *dnaA* gene seems to be still transcribed (Belland et al., 2003). Ongoing DNA replication can be terminated, but not re-initiated due to the lack of DnaA. However, as transcription continues and is even increased for some Trp codon-rich genes (Ouellette et al., 2006; Ouellette et al., 2016), synthesis of some proteins containing no or little Trp is induced (see above) and production of other cell components, especially cell envelope components, can still occur and the ABRs will enlarge.

ATP is required for repair of possible DNA damage in the stalled DNA of the ARBs and it has been shown that both mitochondrial and chlamydial respiratory activities are necessary for ARB formation and maintenance (Liang et al., 2018). Thus, viability of the ARBs is probably maintained by self-produced and host cell-imported ATP (Schmitz-Esser et al., 2004).

Amino acid starvation of *Chlamydia*-infected host cells also leads to ARB formation. It has long been known that bacteria starved for amino acids can complete the ongoing DNA replication cycle, but cannot initiate a new one due to lack of *de novo* DnaA synthesis (Maaloe and Hanawalt, 1961; Lark et al., 1963; Abe and Tomizawa, 1967; Wolf et al., 1968).

An azithromycin-induced *in vitro* persistence model of *C. trachomatis* has been described (Xue et al., 2017) with the generation of ARBs that can revert after removal of azithromycin to infectious EBs. This macrolide antibiotic, as well as erythromycin which also leads to ARB formation (Clark et al., 1982), inhibit protein biosynthesis and hence will again block *de novo* synthesis of DnaA. Penicillin G, as well as other ß-lactam antibiotics, also induces formation of enlarged ARBs (Beatty et al., 1994; Skilton et al., 2009; Kintner et al., 2014). The penicillin-treated chlamydial cells do not divide and continue to enlarge without lysing. DNA replication continues resulting in ARBs with multiple genome copies (Lambden et al., 2006). Removal of penicillin leads to the regeneration of infectious EBs, although the large ARBs do not revert to the normal size RBs. Instead reversion to infectious EB particles occurs by budding RBs from the ARBs which develop to EBs (Skilton et al., 2009). Interestingly, the penicillin-induced ARBs are also resistant to azithromycin (Kintner et al., 2014), suggesting that the additional block of protein biosynthesis exerted by this antibiotic does not further affect the penicillin-induced ARB state. These observations are in accord with the assumption that the penicillin-induced ARBs carry several DNA replication-terminated genomes that are able to re-initiate once the stress condition is removed. This does not occur in the large ARB but rather in single RBs budding from the ARB. In these RBs, re-initiation of DNA replication may take place followed by the normal chlamydial development cycle.

*C. trachomatis* contains a homologue of the universal Obg/CtgA protein (**Table 1**) whose function in the chlamydial intracellular cycle is unknown. This essential GTPase has been shown to trigger persistence in *E. coli* by inducing type I TA modules (see above). A similar Obg-mediated persistence can be excluded for *C. trachomatis* since it lacks TA modules. However, Obg also plays a critical role in several basic cellular processes (Michel, 2005; Kint et al., 2014) including DNA replication by regulating the expression of *dnaA* in *E. coli* (Sikora et al., 2006).

## Conclusions and Perspectives

Persistence is a phenomenon common to most bacteria that probably evolved as a survival strategy against adverse environmental conditions. The formation of persistent human-pathogenic bacteria including IBPs, in particular *M. tubercuslosis*, *Salmonella* serovars, and *Chlamydia* spp., is a very important medical problem linked to chronic infections and to the development of antibiotic resistance. For recent reviews, see (Defraine et al., 2018; Thakur et al., 2019; Wilmaerts et al., 2019). A small subpopulation of persistent cells seems to be already present even in actively growing bacterial populations. These persisters appear to be generated spontaneously by yet unknown process(es). The proportion of persistent members in a bacterial population is increased in the stationary phase suggesting that the persistence trigger is increasingly present in this growth phase. Several stress conditions which the pathogens may encounter during infections, including nutrient starvation accompanied by reduced primary metabolism and energy production, release of the toxin components of various TA modules, stringent stress response with induction of the alarmone (p)ppGpp, general and oxidative stress responses, and especially treatment with various antibiotics, lead to a different but significant increase of the proportion of persistent subpopulations. The antibiotics not only select already existing (and apparently antibiotic-insensitive) persisters in a bacterial cell population, but directly enhance their formation. In fact, treatment with different antibiotics is the most common trigger for the increased incidence of persisters occurring during bacterial infections and also for the generation of persisters *in vitro*. However, none of the above mentioned conditions converts a bacterial population completely into the persistent state, but rather kills the majority of the bacterial population. This indicates that these various processes can stabilize and/or support specifically adapted cells to become peristers, but are not the direct cause of persister formation.

In this review, we cast a new hypothesis into the ongoing debate on the actual mechanism of bacterial persister formation which postulates that the persister state represents the cellular situation in which the DNA replication is terminated, but initiation of a new round of replication is prevented due to an insufficient amount of active ATP-DnaA initiator and possibly by an unaccessible OriC due to positive-supercoiled DNA. Re-initiation of DNA replication requires a precisely regulated amount of ATP-DnaA which depends in part on *de novo* synthesis of DnaA protein and a sufficiently high cellular ATP/ADP ratio. We provide circumstantial evidence that all physiological conditions leading to increased persister formation will also inhibit either DNA replication or protein biosynthesis (and hence *de novo* synthesis of DnaA), or decrease the ATP level thus preventing the formation of the critical cellular concentration of ATP-DnaA necessary for initiation of DNA replication at the newly formed origins of replication. The replication-terminated, but not yet re-initiated chromosomal DNA is in a positive-supercoiled closed circular conformation and hence less sensitive to DNA damage (especially by oxygen and nitrogen radicals) that occurs under virtually all stress conditions that lead to enhanced persister generation.

This mechanism of persister formation could be common to all human bacterial pathogens, even to those intracellular pathogens (e.g., *Chlamydia* and *Rickettsia* spp.) that lack most factors and stress pathways claimed to cause enhanced persistence (mainly identified in *E. coli*). But all bacteria studied so far, including these latter bacterial pathogens, require the ATP-DnaA dependent DNA replication initiation (Kaguni, 2006). As none of the bactericidal antibiotics used to screen for persisters target the ATP-DnaA complex and damage its function (Grimwade and Leonard, 2019), persister cells with terminated DNA replication but stalled initiation are able to re-initiate replication and cell division once the antibiotic is removed.

The correctness of this hypothesis can be experimentally tested and appropriate work is underway to confirm or to falsify it. If it holds true, it opens interesting new opportunities to combat bacterial persister formation.

## Author Contributions

All authors wrote the review. The overall concept and design is based on an idea of WG. All authors contributed to the article and approved the submitted version.

## Funding

We thank the Deutsche Forschungsgemeinschaft for financial support (EI 384/11).

## Conflict of Interest

The authors declare that the research was conducted in the absence of any commercial or financial relationships that could be construed as a potential conflict of interest.
